# Accuracy of transvaginal and transrectal ultrasounds in the diagnosis of endometriosis: A retrospective cohort study

**DOI:** 10.18502/ijrm.v20i5.11051

**Published:** 2022-06-08

**Authors:** Zahra Asgari, Reihaneh Hosseini, Mahdi Sepidarkish, Azar Nabati

**Affiliations:** ^1^Department of Obstetrics and Gynecology, School of Medicine, Tehran University of Medical Sciences, Tehran, Iran.; ^2^Infertility and Reproductive Health Research Center, Health Research Institute, Babol University of Medical Sciences, Babol, Iran.

**Keywords:** Endometriosis, Ultrasonography, Diagnostic imaging, Body mass index.

## Abstract

**Background:**

Early diagnosis and appropriate treatment of endometriosis are vital and may prevent subsequent complications.

**Objective:**

To investigate the diagnostic accuracy of transvaginal ultrasound sonography (TVUS) and transrectal ultrasound sonography for detecting endometriosis considering the age and body mass index (BMI).

**Materials and Methods:**

This was a retrospective cohort study of 119 women scheduled for surgery in a tertiary health care center for clinically suspected endometriosis. Married and virgin women underwent TVUS and transrectal ultrasound sonography, respectively, before laparoscopic excision of endometriotic lesions.

**Results:**

The accuracy of TVUS in the diagnosis of right endometrioma in women with a normal BMI was superior to that in women with a BMI ≥ 30 (95.6% vs. 75.3%; p < 0.001). For the detection of left endometrioma in women with a normal BMI, TVUS demonstrated a sensitivity of 96.9% and a negative predictive value of 92.9%, which was significantly superior to TVUS in women with obesity (sensitivity: 77.4%, negative predictive value: 58.6%). The accuracy of TVUS in the diagnosis of left endometrioma in women under 35 yr was superior to that in women older than 35 yr (93.2% vs. 77.9%; p = 0.04). Similarly, the accuracy of TVUS in the diagnosis of right endometrioma in women under 35 yr was superior to TVUS in women older than 35 yr (86.5% vs. 73.3%; p = 0.04).

**Conclusion:**

Ultrasound can be a useful technique for detecting endometriosis when used adjunctively with the patient's history and physical findings, especially age and BMI.

## 1. Introduction

Endometriosis, which is related to the ectopic endometrial glands and outer stroma of the uterus, is a major gynecological health problem in women of reproductive age, affecting 10-15% of this group (1). Deep endometriosis (where lesions reach a depth of 5 mm), which occurs in 15-30% of all diagnosed cases of endometriosis, can cause symptoms such as cyclical dysmenorrhea, dyschezia, deep dyspareunia, variable digestive complaints, and/or subfertility (2).

Early diagnosis and appropriate treatment are vital and may decrease disease progression and prevent subsequent complications (3). Endometriosis may be suspected by examining the signs and symptoms or by using imaging techniques such as transvaginal ultrasound sonography (TVUS) and magnetic resonance imaging, but the gold standard for diagnosis is laparoscopic identification and histological verification of endometriotic tissue (4). However, due to the invasive nature of laparoscopic identification, non-invasive diagnostic techniques have a higher priority (1). The diagnostic accuracy of TVUS has been assessed in numerous previous studies in various settings and populations with different results. In 2 previous systematic reviews and meta-analyses, although the diagnostic accuracy of TVUS and transrectal ultrasound sonography (TRUS) was estimated as appropriate, high heterogeneity between studies prevented a definitive conclusion (5, 6). As a result of the observed heterogeneity, efforts to further investigate the accuracy of TVUS and TRUS in various situations are reasonable.

The present study aimed to investigate the diagnostic accuracy of TVUS and TRUS for detecting endometriosis considering the age and body mass index (BMI) of participants.

## 2. Materials and Methods 

This retrospective cohort study was carried out from May 2018 and March 2020 in Roointan Arash hospital, a tertiary healthcare center affiliated with Tehran University of Medical Sciences, Tehran, Iran. The hospital is a referral center for endometriosis treatment. Over 2 yr, 119 women who were scheduled for laparoscopic surgery due to signs and symptoms of endometriosis were enrolled. Our inclusion criteria were age > 18 yr and diagnosis of endometriosis based on the symptoms and clinical examination. We excluded those with a history of gynecological surgery or cancer, structural anomalies of the reproductive system, pregnancy, or lack of compliance with TVUS or TRUS. All scans were performed by one of the experienced gynecologists who were blinded to the participants' clinical outcomes.

### Transvaginal sonography

The ultrasound technique used was based on the agreed protocol of the International Deep Endometriosis Analysis group. The review protocol included viewing compartments, peritoneum, and structures in the anterior and posterior parts as well as the uterus and ovaries. We performed TVUS with an Accuvix XQ scanner (Accuvix Sonoace, Medison Co., Ltd, Seoul, South Korea) using a 5-9-MHz probe for transvaginal visualization of the urinary bladder, vagina, adnexal regions, uterus, and uterosacral ligaments. The evaluation was conducted on the non-menstrual days of the cycle. The participants were asked to have a semi-filled bladder and were submitted to a simple rectal enema (fleet enema) 1 hr prior to the procedure. The procedure was done using lubricant gel and without administration of sedatives. As per routine practice, interpretations were done in real-time and documented in printed photographs for future reference. We defined the TVUS diagnosis of endometriosis based on the “presence of regular or irregular hypoechogenic nodular structure or hypoechogenic linear thickening with regular or irregular margins” (7).

### Transrectal sonography

TRUS was performed with an Accuvix XQ scanner (Accuvix Sonoace, Medison Co., Ltd, Seoul, South Korea) using a 5-9-MHz probe for transrectal visualization of the rectosigmoid wall layers. The evaluation was done in non-menstrual days of the cycle. All participants were asked to do the following before the sonography: I) have a soft diet on the day before sonography; II) skip breakfast on the day of the procedure; III) have 2 spoonfuls of milk of magnesium syrup orally after lunch; and IV) take 2 suppositories of 10 mg bisacodyl at 6 PM and 12 midnight on the day before the procedure. The participants were asked to have a semi-filled bladder and were submitted to a simple rectal enema (fleet enema) 1 hr prior to the procedure. The procedure was done using lubricant gel and without administration of sedatives. As per routine practice, interpretations were done in real-time and documented in printed photographs for future reference. We determined the diagnosis of endometriosis based on the presence of regular or irregular hypoechoic nodular structure or hypoechoic linear thickening with regular or irregular margins (7).

### Laparoscopy, radical resection of endometriosis, and histology

**Figure 1:**
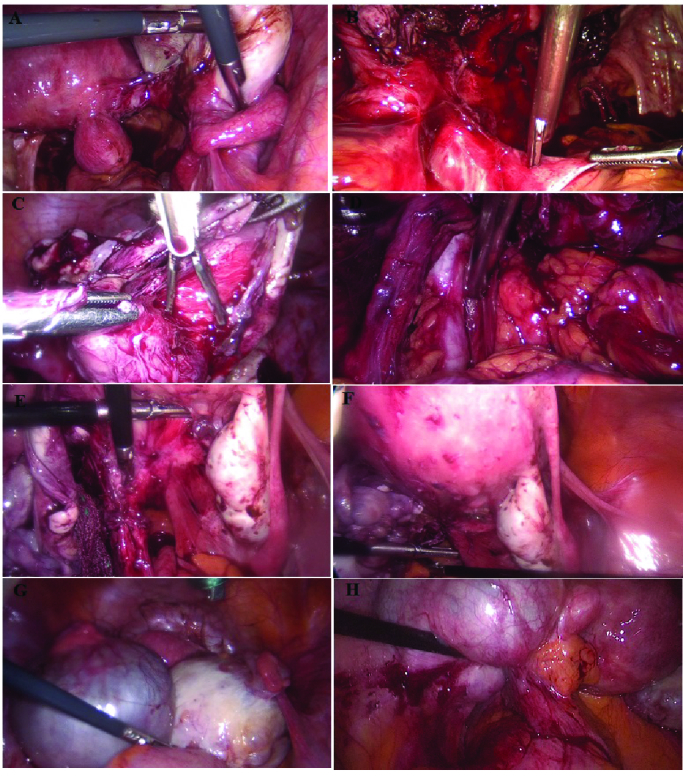
Laparoscopic surgery for diagnosis of infiltrative endometriosis, (A) Right cystectomy of endometrioma, (B) Left ureterolysis, (C) Cystectomy of endometrioma, (D) Resect of nodule of uterosacral, (E) Adhesiolysis in coldosac, (F) Adhesiolysis in coldosac, (G) Enterolysis of rectosigmoid, (H) Adhesiolysis of left ovarian fossa.

All histological confirmations of endometriosis were performed by a pathologist who was blinded to clinical examination and TVUS findings. 2 gynecologists with more than 20 yr experience in radical laparoscopic surgery performed the laparoscopy. We defined deep infiltrating endometriosis as follows: subperitoneal endometriotic infiltration of tissues > 5 mm (Figure 1). All the biopsies were transferred onto a glass slide and appropriately stained by hematoxylin and eosin for microscopic evaluation. An experienced pathologist performed the diagnosis of endometriosis for all resected tissue samples after evaluating both glands and stroma.

### Ethical considerations

Ethics approval was obtained from the Ethical Committee of the Tehran University of Medical Sciences, Tehran, Iran (Code: IR.TUMS.MEDICINE.REC.1399.065). All participants read and signed an informed consent form prior to enrollment in the study. Participants' data were kept confidential and anonymous.

### Statistical analysis

The analyses were carried out using Stata software version 16 (Stata Corp, College Station, Texas, United States). BMI was categorized as underweight ( < 18.5), normal (18.5-24.9), overweight (25-29.9) or obese ( ≥ 30). Continuous variables were described by mean ± standard deviation (SD). Categorical variables were shown as numbers and percentages. We defined accuracy as the results of a diagnosis test (positive or negative) against the true disease using a gold standard (presence or absence). The sensitivity, specificity, positive predictive value (PPV), negative predictive value (NPV), diagnostic odds ratio (DOR), and area under the curve of TVUS and TRUS were evaluated for each involvement site considering BMI and age categories. All accuracy indices were presented with 95% confidence intervals to determine the precision of the results. The accuracy was compared between ultrasound and laparoscopy using McNemar's test. All calculated p-values were 2-tailed. P < 0.05 indicated statistical significance.

## 3. Results

Out of the 168 eligible women, 119 participants were enrolled in this study and 49 were excluded because of having a history of previous surgery for deep infiltrating endometriosis (n = 38), a history of gynecological cancer (n = 5), or because they were not willing to participate in the study (n = 6). The participants' mean ± SD of age was 33.76 ± 7.10 (median: 34 yr; interquartile range: 38, 29), and 26 (21.85%) of them were virgin. Dysmenorrhea was the most common symptom among the participants (91.59%), followed by dyspareunia (52.10%), dyschezia (28.57%), and chronic pelvic pain (10.92%) (Table I).

Endometriosis was histologically confirmed in 117/119 (98.31%, 95% CI: 96.01, 100) women. Histological examination demonstrated that more than 2-3 ${}^{\text{rd}}$ of the women (85 women, 71.42%, 95% CI: 62.42, 79.33) had left endometrioma. The next most common finding was right endometrioma (75/119 women, 63.02%, 95% CI: 53.69, 71.69), followed by pouch of Douglas (68/119, 57.14%, 95% CI: 48.25, 66.03), left ovarian fossa (63/119, 52.94%, 95% CI: 43.57, 62.15), right ovarian fossa (62/119, 52.10%, 95% CI: 43.12, 61.07), left uterosacral ligaments (62/119, 52.10%, 95% CI: 43.12, 61.07), right uterosacral ligaments (55/119, 46.21%, 95% CI: 37.03, 55.59), rectosigmoid (34/119, 28.57%, 95% CI: 20.66, 37.55), and cervix endometriotic nodules (20/119, 16.80%, 95% CI: 10.57, 24.75). Multifocal endometriosis was found in 94 women (78.99%, 95% CI: 70.56, 85.91), which meant that these participants had 2 or more endometriotic nodules affecting the genital, urinary and/or digestive systems (Table II).

### Diagnostic performance of TVUS for the diagnosis of endometriosis

The accuracy, sensitivity, specificity, PPV, NPV, likelihood ratio (LR+) and LR- of TVUS in the prediction of endometriosis involvement at each site are presented in table III. The accuracy of TVUS for the prediction of endometriosis varied between 65.1% (95% CI: 56.7, 73.6), and 92.5% (95% CI: 84.5, 100). The DOR ranged from 3.73 (95% CI: 1.71, 8.11) to 41.5 (95% CI: 12.9, 132) (Table III).

### Diagnostic performance of TRUS for the diagnosis of endometriosis

The sensitivity of TRUS in diagnosing endometriotic lesions in rectosigmoid was 52.9% (95% CI: 35.1, 70.2), the specificity was 94.1% (95% CI: 86.8, 98.1), the PPV was 78.3% (95% CI: 56.3, 92.5), the NPV was 83.3% (95% CI: 74.4, 90.2), the LR+ was 9.01 (95% CI: 3.63, 22.3), the LR- was 0.50 (95% CI: 0.34, 0.71) and the DOR was 18.1 (95% CI: 5.98, 53.7) (Table III).

### Accuracy of TRUS considering age categories

The performance of TVUS in the diagnosis of endometriosis considering age categories is summarized in table IV. The accuracy of TVUS in the diagnosis of left endometrioma in women under 35 yr was superior to that of TVUS in women older than 35 yr (93.2% vs. 77.9%; p = 0.04). Similarly, the accuracy of TVUS in the diagnosis of right endometrioma in women under 35 yr was superior to that of TVUS in women older than 35 yr (86.5% vs. 73.3%; p = 0.04). The accuracy of TVUS for detecting endometriotic lesions or nodules in other sites did not differ between the age categories. TVUS in women under 35 yr seemed to be more specific than TVUS in women above 35 yr in terms of right endometrioma (92.1% vs. 52.6%; p < 0.001) and left uterosacral ligaments (82.9% vs. 65.5%; p = 0.02). Also, the LR+ of TVUS for predicting right endometrioma (10.1 vs. 1.98; p < 0.001) and left endometrioma (10.5 vs. 4.35; p < 0.001) among the women younger than 35 yr were significantly higher than in women older than 35 yr (Table IV).

### Accuracy of TRUS considering age categories

Although the accuracy of TRUS in the diagnosis of rectosigmoid in women older than 35 yr (76.9%, 95% CI: 65.1, 88.7) was superior to in women younger than 35 yr (68.6%, 95% CI: 54.8, 82.4), there was no statistically significant difference between these (p = 0.32).

### Accuracy of TVUS considering BMI categories

The performance of TVUS in the diagnosis of endometriosis considering BMI categories is summarized in table V. The accuracy of TVUS in the diagnosis of right endometrioma (95.6% vs. 75.3%; p < 0.001) in women with a normal BMI was superior to that of TVUS in women with a BMI higher than 30. Also, the LR+ and specificity of TVUS for predicting right endometrioma among women with a normal BMI were superior to those of TVUS in women with a BMI higher than 30. For the detection of left endometrioma in women with a normal BMI, TVUS demonstrated a sensitivity of 96.9%, an NPV of 92.9%, an LR- of 0.03, and a DOR of 215.13, which were significantly superior to TVUS in women with a BMI over 30 (sensitivity: 77.4%, NPV: 58.6%, LR-: 0.25, DOR: 29.1). The accuracy of TVUS for detecting endometriotic lesions or nodules in other sites did not differ between the BMI categories.

### Accuracy of TRUS considering BMI categories

For the detection of the endometriotic lesions in rectosigmoid in women with a normal BMI, TRUS demonstrated a sensitivity of 44.4% (95% CI: 13.7, 78.8), a specificity of 94.7% (95% CI: 82.3, 99.4), a PPV of 66.7% (95% CI: 22.3, 95.7), an NPV of 87.8% (95% CI: 73.8, 95.9), an LR+ of 8.44 (95% CI: 1.82, 39.20), an LR- of 0.59 (95% CI: 0.32, 1.06), and an accuracy of 69.6% (95% CI: 52.1, 87.2), but the differences between these values and those in women with a BMI over 30 were not significant (sensitivity of 56.1% [95% CI: 34.9, 75.6], specificity of 93.6% [95% CI: 82.5, 98.7], PPV of 82.4% [95% CI: 56.6, 96.2], NPV of 80.1% [95% CI: 67.1, 89.6], LR+ of 8.77 [95% CI: 2.78, 27.70], LR- of 0.47 [95% CI: 0.31, 0.73], and accuracy of 74.8% [95% CI: 64.3, 85.3]) (p = 0.53).

**Table I:** The demographic characteristics of the study participants (n = 119)

**Variables**	**Mean ± SD (min, max) (IQR)**	**95% CI**
Age (yr)*	33.76 ± 7.10 (17, 52)	32.47, 35.05
**Body mass index (kg/m**2)*	26.38 ± 3.67 (19.10, 33.98)	25.71, 27.05
Gravidity*	0.98 ± 1.21 (0, 5) (2)	0.76, 1.21
Parity*	0.78 ± 0.97 (0, 4) (1)	0.61, 0.95
Miscarriage*	0.16 ± 0.51 (0, 3) (0)	0.07, 0.26
Stillbirth*	0.03 ± 0.18 (0, 1) (0)	0.01, 0.06
Live birth*	0.79 ± 0.98 (0, 4) (2)	0.61, 0.96
Marital status**
	**Married**	93 (78.15)	69.64, 85.21
	**Virgin**	26 (21.85)	14.79, 30.35
	**Previous underlying disease**	24 (20.16)	13.37, 28.51
	**Previous gynecological surgery**	55 (46.21)	37.03, 55.59
	**Infertility**	34 (28.57)	20.66, 37.57
Presenting symptoms**
	**Dysmenorrhea **	109 (91.59)	85.08, 95.89
	**Dyspareunia **	62 (52.10)	42.75, 61.34
	**Dyschezia **	34 (28.57)	20.66, 37.57
	**Dysuria **	7 (5.88)	2.39, 11.74
	**Chronic pelvic pain **	13 (10.92)	5.94, 17.95

*Data presented as Mean ± SD (min-max). **Data presented as n (%). Min: Minimum, Max: Maximum, 95% CI: 95% confidence interval, IQR: Interquartile range

**Table II:** Location of endometriosis diagnosed by radical resection and histopathological analysis in the 119 suspected endometriosis participants

**Location of endometriosis and histopathologic findings**	**n (%)**	**95% CI**
**Endometrioma (right)**	75 (63.02)	53.69, 71.69
**Endometrioma (left)**	85 (71.42)	62.42, 79.33
**Ovarian fossa (right)**	62 (52.10)	42.75, 61.34
**Ovarian fossa (left)**	63 (52.94)	43.57, 62.15
**Uterosacral ligaments (right)**	55 (46.21)	37.03, 55.59
**Uterosacral ligaments (left)**	62 (52.10)	42.75, 61.34
**Pouch of Douglas**	68 (57.14)	47.74, 66.17
**Vagina**	1 (0.84)	0.02, 4.59
**Cervix**	20 (16.80)	16.80, 24.75
**Rectosigmoid**	34 (28.57)	20.66, 37.57
**Ureter (right)**	1 (0.84)	0.01, 4.59
**Ureter (left)**	2 (1.68)	0.02, 5.93
**Bladder**	2 (1.68)	0.02, 5.93
**Kidney (right) **	5 (4.20)	1.37, 9.53
**Kidney (left) **	5 (4.20)	1.37, 9.53
**Total number of sites affected **	450	
**1 site **	23 (19.32)	12.66, 27.57
**2 sites **	10 (8.41)	4.11, 14.91
**3 sites **	13 (10.92)	5.94, 17.95
**4 sites **	8 (6.72)	2.94, 12.81
**5 sites **	19 (15.96)	9.89, 23.81
**6 sites **	11 (9.24)	4.71, 15.93
**7 sites **	11 (9.24)	4.71, 15.93
**8 sites **	13 (10.92)	5.94, 17.95
**> 9 sites **	9 (7.56)	3.51, 13.87

Data presented as n (%). 95% CI: 95% confidence interval

**Table III:** Diagnostic performance of transvaginal and transrectal sonography for the diagnosis of endometriosis

**Location**	**Sensitivity**	**Specificity**	**PPV**	**NPV**	**Accuracy**	**LR+**	**LR−**	**DOR**
**Endometrioma (right)**	86.7 (76.8, 93.4)	75.1 (59.7, 86.8)	85.5 (75.6, 92.5)	76.7 (61.4, 88.2)	80.8 (73.3, 88.4)	3.47 (2.06, 5.83)	0.17 (0.09, 0.32)	19.50 (7.59, 50.10)
**Endometrioma (left)**	84.7 (75.3, 91.6)	88.2 (72.5, 96.7)	94.7 (87.1, 98.5)	69.8 (53.9, 82.8)	86.5 (79.8, 93.2)	7.20 (2.86, 18.2)	0.17 (0.10, 0.29)	41.50 (12.90, 132.00)
**Ovarian fossa (right)**	82.3 (70.5, 90.8)	71.9 (58.5, 83.1)	76.1 (64.1, 85.7)	78.8 (65.3, 88.9)	77.1 (69.5, 84.7)	2.93 (1.90, 4.51)	0.24 (0.14, 0.43)	11.90 (5.01, 28.20)
**Ovarian fossa (left)**	85.7 (74.6, 93.3)	71.4 (57.8, 82.7)	77.1 (65.6, 86.3)	81.6 (68.1, 91.2)	78.6 (71.2, 86.1)	3.01 (1.96, 4.59)	0.21 (0.10, 0.37)	15.10 (6.07, 37.10)
**Uterosacral ligaments (right)**	63.6 (49.6, 76.2)	75.1 (62.6, 85.1)	68.6 (54.1, 80.9)	70.6 (58.3, 81.1)	69.3 (61.1, 77.7)	2.55 (1.59, 4.07)	0.48 (0.33, 0.70)	5.25 (2.41, 11.50)
**Uterosacral ligaments (left)**	54.8 (41.7, 67.5)	75.4 (62.2, 85.9)	70.8 (55.9, 83.1)	60.6 (48.3, 72.1)	65.1 (56.7, 73.6)	2.23 (1.34, 3.71)	0.59 (0.43, 0.81)	3.73 (1.71, 8.11)
**Pouch of Douglas**	75.1 (63.1, 84.7)	68.6 (54.1, 80.9)	76.1 (64.1, 85.7)	67.3 (52.9, 79.7)	71.8 (63.6, 80.1)	2.39 (1.56, 3.67)	0.36 (0.23, 0.57)	6.56 (2.94, 14.60)
**Cervix**	85.1 (62.1, 96.8)	100 (96.3, 100)	100 (80.5, 100)	97.1 (91.6, 99.4)	92.5 (84.5, 100)	NE	0.15 (0.05, 0.42)	NE
**Rectosigmoid**	52.9 (35.1, 70.2)	94.1 (86.8, 98.1)	78.3 (56.3, 92.5)	83.3 (74.4, 90.2)	73.5 (64.7, 82.4)	9.01 (3.63, 22.3)	0.50 (0.34, 0.71)	18.10 (5.98, 53.70)

Data presented as point estimate and corresponding 95% CI. NE: Not estimated, PPV: Positive predictive value, NPV: Negative predictive value, LR: Likelihood ratio, DOR: Diagnostic odds ratio

**Table IV:** Diagnostic performance of transvaginal sonography for the diagnosis of endometriosis considering age categories

**Location**	**Age (yr)**	**Sensitivity**	**Specificity**	**PPV**	**NPV**	**Accuracy (%)**	**LR+**	**LR−**	**DOR**
**Endometrioma (right)**	≤ 35	81.1 (65.9, 91.4)	92.1 (74.1, 99.1)	94.4 (81.3, 99.3)	74.2 (55.4, 88.1)	86.5 (78.4, 94.6)	10.10 (2.66, 38.5)	0.20 (0.11, 0.39)	48.90 (10.30, 85.30)
	> 35	93.9 (79.8, 99.3)	52.6 (28.9, 75.6)	77.5 (61.5, 89.2)	83.3 (51.6, 97.9)	73.3 (61.1, 85.5)	1.98 (1.22, 3.21)	0.11 (0.02, 0.47)	17.20 (3.46, 28.60)
**Endometrioma (left)**	≤ 35	95.6 (84.9, 99.5)	90.9 (70.8, 98.9)	95.6 (84.9, 99.5)	90.9 (70.8, 98.9)	93.2 (86.4, 100)	10.50 (2.80, 39.50)	0.05 (0.01, 0.19)	215.00 (30.60, 1513.00)
	> 35	72.5 (56.1, 85.4)	83.3 (51.6, 97.9)	93.5 (78.6, 99.2)	47.6 (25.7, 70.2)	77.9 (64.9, 91.1)	4.35 (1.21, 15.60)	0.13 (0.18, 0.58)	13.20 (2.71, 32.10)
**Ovarian fossa (right)**	≤ 35	74.2 (55.4, 88.1)	77.8 (60.8, 89.9)	74.2 (55.4, 88.1)	77.8 (60.8, 89.9)	76.1 (65.6, 86.4)	3.34 (1.75, 6.37)	0.33 (0.18, 0.62)	10.10 (3.32, 30.50)
	> 35	90.3 (74.2, 98.1)	61.9 (38.4, 81.9)	77.8 (60.8, 89.9)	81.3 (54.4, 96.1)	76.1 (64.2, 88.1)	2.37 (1.36, 4.14)	0.15 (0.05, 0.48)	15.20 (3.61, 62.30)
**Ovarian fossa (left)**	≤ 35	79.3 (60.3, 92.1)	73.7 (56.9, 86.6)	69.7 (51.3, 84.4)	82.4 (65.5, 93.2)	76.5 (66.2, 86.8)	3.01 (1.72, 5.31)	0.28 (0.13, 0.58)	10.70 (3.45, 33.3)
	> 35	91.2 (76.3, 98.1)	66.7 (41.1, 86.7)	83.8 (68.1, 93.8)	80.1 (51.9, 95.7)	78.9 (66.7, 91.1)	2.74 (1.41, 5.31)	0.13 (0.04, 0.41)	20.70 (4.65, 90.40)
**Uterosacral ligaments (right)**	≤ 35	59.4 (40.6, 76.3)	82.9 (66.4, 93.4)	76.1 (54.9, 90.6)	69.1 (52.9, 82.4)	71.1 (60.4, 81.8)	3.46 (1.58, 7.58)	0.49 (0.31, 0.76)	7.06 (2.33, 21.30)
	> 35	69.6 (47.1, 86.8)	65.5 (45.7, 82.1)	61.5 (40.6, 79.8)	73.1 (52.2, 88.4)	67.5 (54.5, 80.6)	2.02 (1.14, 3.57)	0.46 (0.23, 0.91)	4.34 (1.37, 13.80)
**Uterosacral ligaments (left)**	≤ 35	50.1 (31.9, 68.1)	85.7 (69.7, 95.2)	76.2 (52.8, 91.8)	65.2 (49.8, 78.6)	67.9 (57.3, 78.4)	3.51 (1.45, 8.46)	0.58 (0.40, 0.84)	6.01 (1.91, 18.70)
	> 35	60.1 (40.6, 77.3)	59.1 (36.4, 79.3)	66.7 (46.1, 83.5)	52.1 (31.3, 72.2)	59.5 (45.8, 73.3)	1.47 (0.82, 2.62)	0.68 (0.38, 1.18)	2.17 (0.71, 6.64)
**Pouch of Douglas**	≤ 35	69.7 (51.3, 84.4)	64.7 (46.5, 80.3)	65.7 (47.8, 80.9)	68.8 (50.1, 83.9)	67.2 (55.8, 78.6)	1.97 (1.19, 3.28)	0.47 (0.26, 0.83)	4.22 (1.53, 11.60)
	> 35	80.1 (63.1, 91.6)	76.5 (50.1, 93.2)	87.5 (71.1, 96.5)	65.1 (40.8, 84.6)	78.2 (65.9, 90.6)	3.41 (1.42, 8.14)	0.26 (0.12, 0.53)	13.10 (3.33, 50.30)
**Cervix**	≤ 35	75.1 (34.9, 96.8)	100 (93.9, 100)	100 (54.1, 100)	96.7 (88.7, 99.6)	87.5 (71.5, 100)	NE	0.25 (0.07, 0.83)	NE
	> 35	91.7 (61.5, 99.8)	100 (91.2, 100)	100 (71.5, 100)	97.6 (87.1, 99.9)	95.8 (87.7, 100)	NE	0.08 (0.01, 0.54)	NE
**Rectosigmoid**	≤ 35	42.9 (17.7, 71.1)	94.3 (84.3, 98.8)	66.7 (29.9, 92.5)	86.2 (74.6, 93.9)	68.6 (54.8, 82.4)	7.52 (2.16, 26.50)	0.61 (0.38, 0.95)	12.50 (2.78, 55.70)
	> 35	60.1 (36.1, 80.9)	93.8 (79.2, 99.2)	85.7 (57.2, 98.2)	78.9 (62.7, 90.4)	76.9 (65.1, 88.7)	9.61 (2.39, 38.5)	0.42 (0.24, 0.73)	22.50 (4.51, 49.70)

Data presented as point estimate and corresponding 95% CI. NE: Not estimated, PPV: Positive predictive value, NPV: Negative predictive value, LR: Likelihood ratio, DOR: Diagnostic odds ratio

**Table V:** Diagnostic performance of transvaginal sonography for the diagnosis of endometriosis considering weight categories

**Location**	**BMI**	**Sensitivity**	**Specificity**	**PPV**	**NPV**	**Accuracy (%)**	**LR+**	**LR−**	**DOR**
**Endometrioma (right)**	Normal	80.8 (60.6, 93.4)	90.5 (69.6, 98.8)	91.3 (72.1, 98.9)	79.2 (57.8, 92.9)	95.6 (85.6, 99.7)	8.48 (2.24, 32.10)	0.21 (0.09, 0.47)	39.9 (7.42, 206.00)
	Overweight and obese	89.8 (77.8, 96.6)	60.9 (38.5, 80.3)	83.1 (70.2, 91.9)	73.7 (48.8, 90.9)	75.3 (64.3, 86.4)	2.29 (1.37, 3.85)	0.16 (0.06, 0.40)	13.70 (4.03, 46.30)
**Endometrioma (left)**	Normal	96.9 (83.8, 99.9)	86.7 (59.5, 98.3)	93.9 (79.8, 99.3)	92.9 (66.1, 99.8)	91.8 (82.4, 100)	7.27 (2.10, 26.4)	0.03 (0.01, 0.25)	215.00 (30.60, 1513.00)
	Overweight and obese	77.4 (63.8, 87.7)	89.5 (66.9, 98.7)	95.3 (84.2, 99.4)	58.6 (38.9, 76.5)	83.4 (74.3, 92.5)	7.35 (1.97, 27.50)	0.25 (0.15, 0.42)	29.10 (6.38, 40.10)
**Ovarian fossa (right)**	Normal	75.1 (50.9, 91.3)	77.8 (57.7, 91.4)	71.4 (47.8, 88.7)	80.8 (60.6, 93.4)	76.4 (63.8, 89.1)	3.38 (1.59, 7.14)	0.32 (0.14, 0.70)	10.50 (2.77, 39.80)
	Overweight and obese	85.7 (71.5, 94.6)	66.7 (47.2, 82.7)	78.3 (63.6, 89.1)	76.9 (56.4, 91.1)	76.2 (66.1, 86.3)	2.57 (1.53, 4.33)	0.21 (0.09, 0.46)	12.10 (3.87, 37.10)
**Ovarian fossa (left)**	Normal	78.9 (54.4, 93.9)	75.1 (55.1, 89.3)	68.2 (45.1, 86.1)	84.1 (63.9, 95.5)	77.1 (64.5, 89.4)	3.16 (1.61, 6.25)	0.28 (0.11, 0.69)	11.30 (2.87, 43.70)
	Overweight and obese	88.6 (75.4, 96.2)	67.9 (47.6, 84.1)	81.3 (67.4, 91.1)	79.2 (57.8, 92.9)	78.2 (68.2, 88.3)	2.76 (1.59, 4.77)	0.16 (0.07, 0.39)	16.50 (4.96, 54.40)
**Uterosacral ligaments (right)**	Normal	60.1 (36.1, 80.9)	85.2 (66.3, 95.8)	75.1 (47.6, 92.7)	74.2 (55.4, 88.1)	72.6 (59.6, 85.6)	4.05 (1.53, 10.7)	0.47 (0.27, 0.82)	8.63 (2.22, 33.10)
	Overweight and obese	65.7 (47.8, 80.9)	67.6 (50.2, 80.1)	65.7 (47.8, 80.9)	67.6 (50.2, 80.1)	66.6 (55.6, 77.7)	2.03 (1.21, 3.42)	0.50 (0.30, 0.84)	3.99 (1.51, 10.50)
**Uterosacral ligaments (left)**	Normal	45.1 (23.1, 68.5)	77.8 (57.7, 91.4)	60.1 (32.3, 83.7)	65.6 (46.8, 81.4)	61.4 (47.6, 75.1)	2.02 (0.86, 4.77)	0.71 (0.45, 1.01)	2.86 (0.83, 9.86)
	Overweight and obese	59.5 (43.3, 74.4)	73.3 (54.1, 87.7)	75.8 (57.7, 88.9)	56.4 (39.6, 72.2)	66.4 (55.4, 77.4)	2.23 (1.17, 4.25)	0.55 (0.36, 0.84)	4.04 (1.48, 11.00)
**Pouch of Douglas**	Normal	72.7 (49.8, 89.3)	68.1 (46.5, 85.1)	66.7 (44.7, 84.4)	73.9 (51.6, 89.8)	70.4 (57.1, 83.7)	2.27 (1.22, 4.25)	0.40 (0.19, 0.83)	5.67 (1.64, 19.50)
	Overweight and obese	76.1 (61.2, 87.4)	69.2 (48.2, 85.7)	81.4 (66.6, 91.6)	62.1 (42.3, 79.3)	72.7 (61.7, 83.6)	2.47 (1.36, 4.51)	0.34 (0.19, 0.61)	7.16 (2.48, 20.60)
**Cervix**	Normal	100 (54.1, 100)	100 (91.4, 100)	100 (54.1, 100)	100 (91.4, 100)	100 (100, 100)	NE	NE	NE
	Overweight and obese	78.6 (49.2, 95.3)	100 (93.8, 100)	100 (71.5, 100)	95.1 (86.3, 99.1)	89.3 (78.1, 100)	NE	NE	NE
**Rectosigmoid**	Normal	44.4 (13.7, 78.8)	94.7 (82.3, 99.4)	66.7 (22.3, 95.7)	87.8 (73.8, 95.9)	69.6 (52.1, 87.2)	8.44 (1.82, 39.2)	0.59 (0.32, 1.06)	14.40 (2.35, 86.90)
	Overweight and obese	56.1 (34.9, 75.6)	93.6 (82.5, 98.7)	82.4 (56.6, 96.2)	80.1 (67.1, 89.6)	74.8 (64.3, 85.3)	8.77 (2.78, 27.70)	0.47 (0.31, 0.73)	18.70 (4.78, 71.30)

Data presented as point estimate and corresponding 95% CI. NE: Not estimated, PPV: Positive predictive value, NPV: Negative predictive value, LR: Likelihood ratio, DOR: Diagnostic odds ratio, BMI: Body mass index, Normal weight: BMI: 18.5-24.9, Overweight: BMI: 25-29.9, Obesity: BMI > 30

## 4. Discussion

The findings of this study demonstrated 3 points: first, ultrasound was an accurate diagnostic method for the assessment of women with suspected endometriosis; second, the accuracy of TVUS in the diagnosis of endometrioma and uterosacral ligaments endometriosis in women with normal weight was higher than in overweight or obese women; third, the accuracy of TVUS in the diagnosis of endometrioma and uterosacral ligaments endometriosis in women under 35 yr was superior to that of TVUS in women older than 35 yr. The first of these findings is comparable to those reported by previous studies.

A systematic review and meta-analysis by Guerriero and colleagues evaluated the evidence from previous primary studies on the diagnostic accuracy of TVUS in the preoperative detection of endometriosis. A comprehensive search was performed in 2 prominent databases for studies published between January 1989 and December 2014, and finally, 11 studies (n = 1,583) were considered eligible and were included in the meta-analysis. “Meta-analysis of 11 studies (including 1,583 women) revealed a pooled sensitivity of 53% (95% CI: 35, 70) and specificity of 93% (95% CI: 83, 97) with the SROC equal to 0.97 (95% CI 0.95, 0.98) detection of endometriosis in the uterosacral ligaments. The overall pooled sensitivity and specificity were 49% (95% CI: 36, 62%) and 98% (95% CI: 95, 99%), for detection of endometriosis in the rectovaginal septum. The summary estimates were 58% (95% CI: 40, 74%) for sensitivity and 96% (95% CI: 87, 99%) for specificity for detection of vaginal endometriosis. For detection of bladder endometriosis, the overall pooled sensitivity and specificity were 62% (95% CI: 40, 80%) and 100% (95% CI: 97, 100%), respectively. Substantial heterogeneity was found for sensitivity and specificity for all these locations. The authors conclude that the overall diagnostic performance of TVUS for detecting endometriosis in uterosacral ligaments, rectovaginal septum, vagina, and the bladder is fair with high specificity. They emphasized that the study failed to investigate the source of heterogeneity and due to this observed heterogeneity, the need for an international consensus is essential to create future prospective multicenter studies and improve further the methodology” (5).

In another systematic review and meta-analysis that was performed by the same investigators, studies published between January 1989 and December 2014 were searched. “An extended search identified a total of 801 citations, among which 19 studies (n = 2639) were considered eligible and included in the meta-analysis. Overall pooled sensitivity, specificity, LR+ and LR- of TVUS for detecting endometriosis in the rectosigmoid were 91% (95% CI: 85, 94%), 97% (95% CI: 95, 98%), 33.0 (95% CI: 18.6, 58.6) and 0.10 (95% CI: 0.06, 0.16), respectively. Similarly, considerable heterogeneity was found for accuracy indices” (6). Nearly all of the samples of the studies that were included that assessed the accuracy of ultrasound for detecting endometriosis were of women of reproductive age with a weight of normal range. Obesity can affect the diagnostic accuracy of ultrasound in 2 ways: 1) increasing the thickness of the body organs and consequently reducing the penetration depth of the ultrasound beam; and 2) decreasing the depth of ultrasound permeability to subcutaneous and intra-peritoneal fat (8). Adipose tissue reduces the penetration rate of ultrasound radiation by 0.63 dB/cm. It also absorbs and disperses the ultrasound beam, resulting in an inappropriate assessment of the organs of the reproductive system (9).

With experience and awareness, clinical diagnosis has probably improved since the studies that were included in those systematic reviews were published. Data from comparative studies suggest that women's characteristics might influence the effectiveness of the diagnostic workup and ultrasound accuracy by giving a different prior-test probability of endometriosis (10). The results of a national case-control study by Ballard and colleagues, showed that “the specific symptoms and frequent medical consultation are associated with endometriosis and the likelihood of endometriosis increased with the number of symptoms present, from an odds ratio of 5.0 with one symptom to 84.7 for 7 or more symptoms” (11).

Several investigators have used this approach to develop models for predicting endometriosis (11, 12). In another study by Lafay Pillet and colleagues, a diagnostic score of endometriosis associated with endometriomas using 4 clinical symptoms was developed. “4 variables were: visual analogue scale of gastro-intestinal symptoms ≥ 5 or of deep dyspareunia > 5 (adjusted diagnostic odds ratio (aDOR) = 6.0, 95% CI: [2.9, 12.1]), duration of pain greater than 24 months (aDOR = 3.8, 95% CI: [1.9, 7.7]), severe dysmenorrhea (defined as the prescription of the oral contraceptive pill for the treatment of a primary dysmenorrhea or the worsening of a secondary dysmenorrhea) (aDOR = 3.8, 95% CI: [1.9,7.6]) and primary or secondary infertility (aDOR = 2.5, 95% CI: [1.2, 4.9])” (13).

### Limitations

There were several limitations in this study that need to be discussed. First, the study was performed in a referral center for the treatment of gynecological diseases; therefore, the probability of endometrial lesions in the study population was high, representing a selection bias. So, we cannot extrapolate the findings to the general population of women with clinical suspicion of endometriosis. Second, the sonographer was aware of the findings of the preoperative clinical examination. Third, TVUS examinations were performed by an experienced sonographer; therefore, these results may not be repeated by an inexperienced sonographer. Fourth, there was a low frequency of bladder, ureter, and vagina endometriosis, which increased the random error, and it was not possible to calculate diagnostic indicators.

## 5. Conclusion

In conclusion, ultrasound can be a useful technique for detecting endometriosis when used adjunctively with the patient's history and physical findings, especially age and BMI.

##  Conflict of Interest

The authors declare that there is no conflict of interest.
